# Supplementary material for: Identifying repeat domains in large genomes
Source: Genome Biol. 2006 Jan 31;7(1):R7. doi: 10.1186/gb-2006-7-1-r7 (PMC1431705; doi:10.1186/gb-2006-7-1-r7)
Supplement: Additional File 1 — A zipped file of browsable HTML files with a complete list of the connected components in the repeat domain graph of human Repbase. [file gb-2006-7-1-r7-S1.gz › html/subgraphs/8072.pdf]

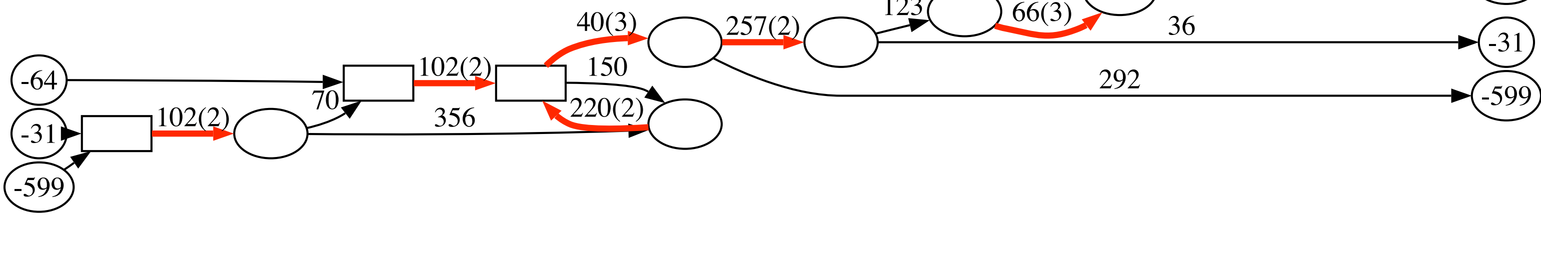

-592
108
425
-64
24
-592
96(2)
126
114(2)
66(3)
123
40(3)
257(2)
36
-31
-64
102(2)
150
292
-599
-31
102(2)
70
356
220(2)
-599
